# Spatial Heterogeneity in the Occurrence Probability of Rainstorms over China

**DOI:** 10.3390/e20120958

**Published:** 2018-12-12

**Authors:** Yan-Fang Sang

**Affiliations:** Key Laboratory of Water Cycle and Related Land Surface Processes, Institute of Geographic Sciences and Natural Resources Research, Chinese Academy of Sciences, Beijing 100101, China; sangyf@igsnrr.ac.cn or

**Keywords:** rainstorm, water disasters, spatiotemporal variability, entropy, occurrence probability

## Abstract

Detecting the spatial heterogeneity in the potential occurrence probability of water disasters is a foremost and critical issue for the prevention and mitigation of water disasters. However, it is also a challenging task due to the lack of effective approaches. In the article, the entropy index was employed and those daily rainfall data at 520 stations were used to investigate the occurrences of rainstorms in China. Results indicated that the entropy results were mainly determined by statistical characters (mean value and standard deviation) of rainfall data, and can categorically describe the spatial heterogeneity in the occurrence of rainstorms by considering both their occurrence frequencies and magnitudes. Smaller entropy values mean that rainstorm events with bigger magnitudes were more likely to occur. Moreover, the spatial distribution of entropy values kept a good relationship with the hydroclimate conditions, described by the aridity index. In China, rainstorms are more to likely occur in the Pearl River basin, Southeast River basin, lower-reach of the Yangtze River basin, Huai River basin, and southwest corner of China. In summary, the entropy index can be an effective alternative for quantifying the potential occurrence probability of rainstorms. Four thresholds of entropy value were given to distinguish the occurrence frequency of rainstorms as five levels: very high, high, mid, low and very low, which can be a helpful reference for the study of daily rainstorms in other basins and regions.

## 1. Introduction

Water disasters, triggered by rainstorms, have been becoming a major type of natural hazard worldwide [1,2], and have thus received extensive attention in recent times. Over the last decade, rainstorms and water disasters increasingly occur in many basins and regions [3], especially in July and August of every year in monsoon-affected areas. In China, the annual human fatalities (>300) and economic losses (>US$40 billion) caused by water-related disasters occupy more than half of the total losses from all types of natural disasters [4].

Due to obvious spatial heterogeneity in the geographic and hydroclimate conditions, the occurrence frequencies of rainstorms and water disasters and their magnitudes vary with regions in China [5,6]. To formulate proactive and effective adaptation strategies for the control of water disasters, it is a critical issue to accurately identify and regionalize the key regions where water disasters with high magnitudes frequently occur. However, rainstorms and water disasters usually occur on short (hourly, daily and weekly) time scales and in local regions, where hydroclimate stations are scattered or even missing, causing difficulties in obtaining enough observed data. The drastic spatiotemporal variability of water disasters cannot be easily captured from the limited observed data. Therefore, it is still a challenging task to accurately detect the spatial heterogeneity in the potential occurrence probability of water disasters.

To meet the need, many indices have been proposed and used to quantify the potential occurrence probability of water disasters, whose intensity, frequency and duration are usually considered together [7,8]. The occurrence frequency and (both total and average) magnitude are commonly used two indices [3]. Statistical characters (mean value, variation coefficient, skewness coefficient, etc.) of hydroclimate data are also widely considered to describe the spatiotemporal variability of water disasters [9]. However, each index can only describe certain characteristics of water disasters, and the values of various indices usually have different spatial distributions [10], causing confusion of the spatial heterogeneity in the occurrence probability of water disasters. When using some indices together to investigate the occurrence probability of water disasters, the results cannot be easily integrated to get a uniform conclusion. Thereby, more effective indexes which can synthetically reflect the results of different indices should be established to meet the urgent needs.

In information theory, the entropy index is a primary measure of uncertainty degree, such as disorderliness, randomness, and irregularity [11,12]. Higher entropy reflects more random system and less useful information in it. In contrast, for those systems with lower entropy values, they include more useful information and show more regular variability. Information theory has been widely applied in hydrology for detecting hydrological variability [13], derivation of hydrological distributions, estimation of models’ parameters and uncertainty [14,15], and others [16,17]. Here, the main objective is to explore whether the index of entropy can be an effective measure for quantifying the spatial heterogeneity in the occurrence probability of rainstorms, as an important indicator of water disasters.

## 2. Data and Methods

The daily rainfall data measured at 520 meteorological stations during 1961–2013 are used to analyze rainstorms in China. These meteorological stations are more distributed in the out-flowing river regions in the mid and southeast part of China, where rainstorms and water disasters occur frequently under the effects of Southeast Asian monsoon and tropical cyclones. Those dry days (with a magnitude smaller than 0.1 mm) in the daily rainfall data at each station are removed first to eliminate their influence on the analysis of rainstorms [18], and the residual values are called daily rainy data (DRD).

The Shannon entropy index, with an explicit definition and being easily calculated [19], is used to quantify the statistical characters of DRD. The Shannon entropy (*E*) can be calculated as:

*E*(*x*) = ∑[*f*(*x_i_*)log_2_(*f*(*x_i_*))], (1)

where *f*(*x_i_*) is the probability density function (PDF) used to describe the random characteristics of the DRD variable *x* with the length of *n* (*i* = 1, 2, …, *n*). Then, the relationship between the E values and the statistical characters of DRD, as well as the occurrence frequencies and magnitudes of different rainfall extremes, are investigated to explore the efficiency of the entropy index. The spatial difference in hydroclimate conditions, quantified by the aridity index (AI), is considered to explain the physical causes of the spatial distribution of entropy values. The AI is defined as the ratio of annual precipitation to annual potential evaporation at each station [20], and it can reflect the average water budget conditions in a region.

## 3. Results and Discussion

**Figure 1:**
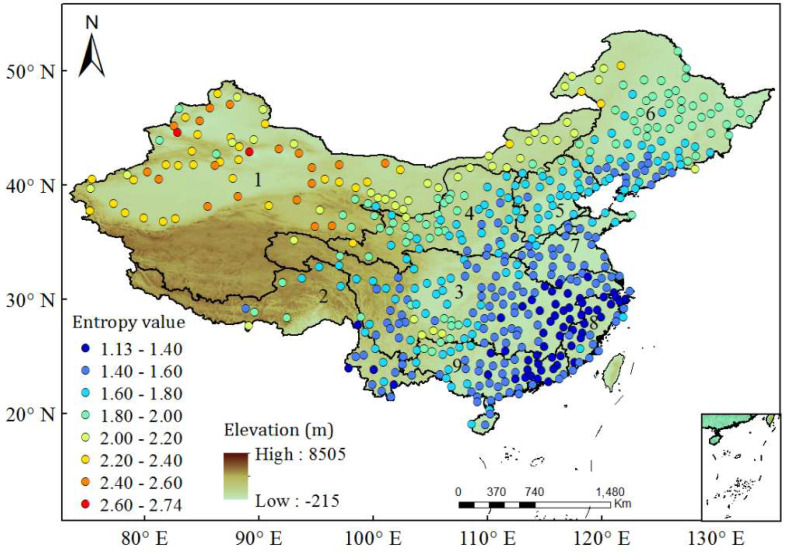
Spatial distribution of the entropy values of daily rainfall data in China. Dry days in the daily rainfall data at each station are removed and then the residual is used to calculate entropy values. The daily rainfall data measured at 520 meteorological stations in China are considered here. 1, the Northwest Inland River basin; 2, the Southwest River basin; 3, the Yangtze River basin; 4, the Yellow River basin; 5, the Haihe River basin; 6, the Songliao River basin; 7, the Huaihe River basin; 8, the Southeast River basin; and 9, the Pearl River basin.

Figure 1 presents the spatial distribution of the entropy values of DRD in China, with a regular increasing pattern from southeast to northwest. DRD has an entropy value of 1.1~1.6 in the southeast part of China, including the mid- and lower-reaches of the Pearl River basin, the Southeast River basin, the lower-reach of the Yangtze River basin, and the Huai River basin. In the semi-arid and semi-moist transition zones coving the Songliao River basin, the Hai River basin, and the mid-reaches of the Yellow and Yangtze River basins, DRD has an entropy value of 1.6~2.0. There is a special local region in the up-reach of the Pearl River basin in Southwest China, where DRD has relatively bigger entropy value compared with the surrounding regions. Comparatively, DRD has an entropy value bigger than 2.0 in Northwest China. The spatial distribution of the entropy values of DRD on the whole accords well with the hydrographic features in China, which are determined by the typical terrain conditions, i.e., high in the West and low in the East, and the climate conditions of the Southeast Asian monsoon [21].

**Figure 2:**
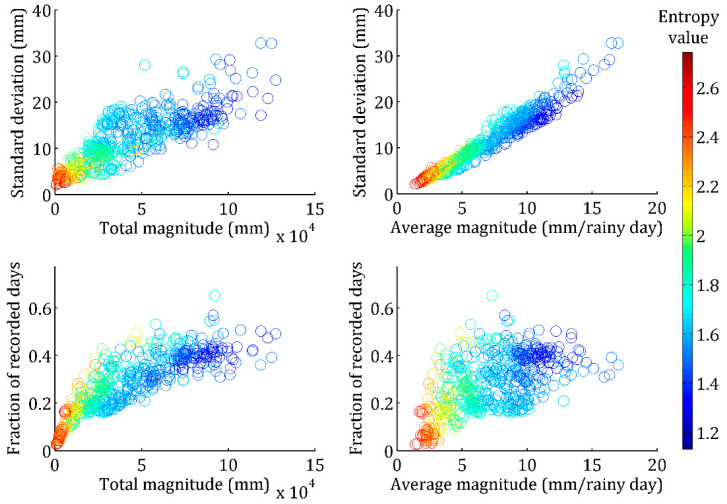
Relationship between the entropy values and statistical characters (standard deviation and total (average) magnitude) of daily rainfall data, and the rainy days (occurrence times) and its magnitudes.

To verify the efficiency of the entropy index, the relationship between the entropy value and the statistical characters (standard deviation and average magnitude) of DRD, as well as its sample sizes (i.e., fraction of recorded days) and total magnitude at each station, are analyzed. Results indicate that the entropy value has no relationship with skew characteristics of DRD, and thus, it is omitted here. Interestingly, Figure 2 shows that along with the increase of the standard deviation value and magnitude (especially average magnitude) of DRD, the entropy value correspondingly decreases. Entropy value of DRD also decreases with the increase of its occurrence times. As a result, it was found that the entropy value may be determined by statistical characters (especially the mean value and standard deviation) of DRD, but not by either of these statistical factors alone. Thus, the index can synthetically reflect the occurrence of rainy days and rainfall magnitudes. Small entropy values indicate that rainfall events with big magnitudes are more likely to occur, and so reflect more obvious variation in the rainfall process.

**Figure 3:**
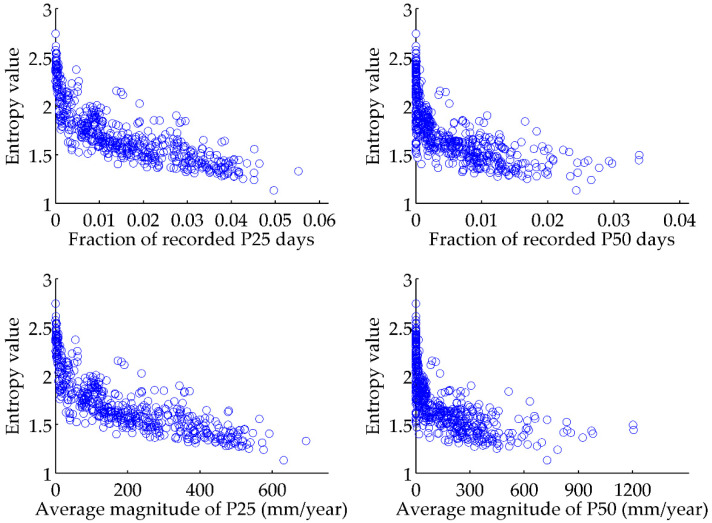
Relationship between the entropy values and occurrence times and total magnitude of the P25 and P50 rainstorms. P25 rainstorm means the daily rainfall data with a magnitude between 25 mm and 50 mm; P50 rainstorm means the daily rainfall data with a magnitude bigger than 50 mm.

**Figure 4:**
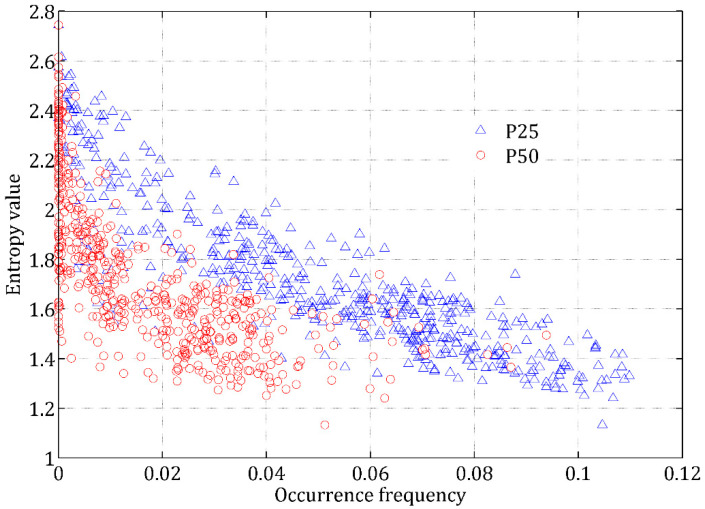
Relationship between the entropy values and occurrence frequency (occurrence times divided by total rainy days) of P25 and P50 rainstorms. P25 rainstorm means the daily rainfall data with a magnitude between 25 mm and 50 mm; P50 rainstorm means the daily rainfall data with a magnitude bigger than 50 mm.

To further verify the efficiency of the entropy index to present the potential occurrence of rainstorms, two ranks of rainstorms, with a daily magnitude of 25~50 mm (denoted as P25) and bigger than 50 mm (denoted as P50), are further considered here. P25 and P50 are commonly used to quantify the intensities of different rainstorms in China [22], and their relationship with the entropy value of DRD is analyzed. In Figure 3, both the occurrence times (i.e., fraction of recorded days) and average magnitude of the P25 and P50 rainstorms consistently increase with entropy value decrease. The occurrence frequencies (occurrence times divided by total rainy days) of P25 and P50 rainstorms also increase with the decrease of entropy values (Figure 4). Therefore, smaller entropy values mean that more rainstorm events with bigger magnitudes are more likely to occur, being consistent with the results in Figure 2. Based on the spatial distribution of entropy values in Figure 1, it is known that in the southeast part of China with smallest entropy values, rainstorm events are more likely to occur; however, rainstorms are not likely to occur in Northwest China.

**Figure 5:**
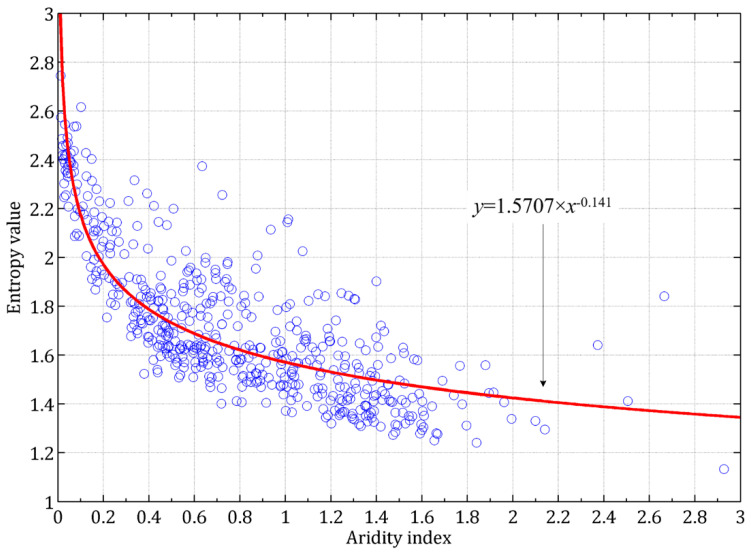
Relationship between the entropy values and the values of the aridity index (AI), which basically reflects the hydroclimate condition and average water budget in a region.

Considering that the occurrence frequencies of rainstorms should be physically related to the hydroclimate conditions, especially in those monsoon-affected regions in China, it is verified here by analyzing the relationship between the entropy values and the AI values. Their good negative relationship is established and can be described by the power function of *y* = 1.5707 × *x*^−0.141^, with the determination coefficient of 0.70 (Figure 5). Therefore, it is thought that although the occurrences of rainstorm events are caused by specific physical mechanisms and have different magnitudes and potential influences, the occurrence frequencies of rainstorms in a certain region is overall determined by its background of hydroclimate conditions, and those regions with more positive water budget conditions would have higher occurrence probabilities of rainstorms. From the results, it is known that the entropy results have reliable hydroclimate bases, and the entropy index is an effective measure to present the occurrence probability of rainstorms and its spatial heterogeneity.

**Figure 6:**
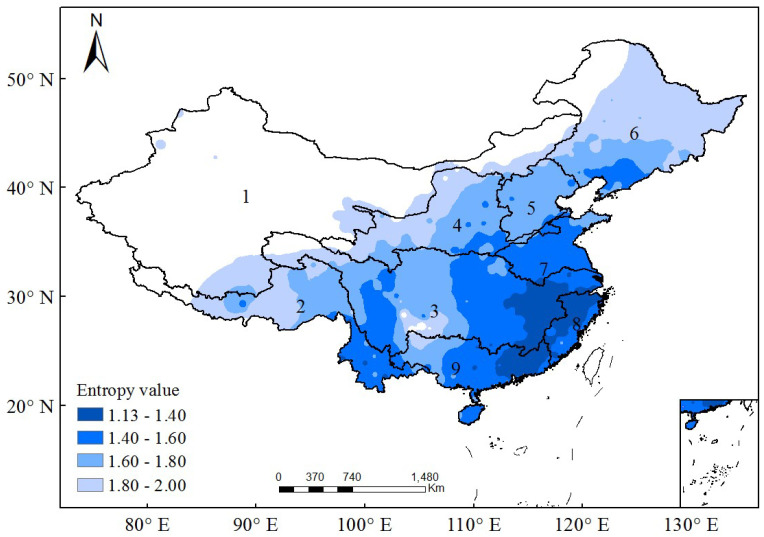
Entropy-based classification of those regions with a very high, high, mid and low occurrence probability of rainstorms in China.

In order to distinguish the regions where rainstorm events have different potential occurrence probabilities, the matching condition between the spatial distribution of entropy values and the locations of nine major basins in China is mainly considered, and then the value range of entropy is basically divided into five segments as <1.4, 1.4~1.6, 1.6~1.8, 1.8~2.0 and >2.0, corresponding to very high, high, mid, low, and very low probabilities of the potential occurrence of rainstorms and water disasters in China. The four thresholds of entropy value correspond to the AI values of 2.26, 0.88, 0.38, and 0.18, basically reflecting the hyper-humid, humid, semiarid, and arid conditions, respectively. The regions corresponding to the five entropy value ranges are shown in Figure 6. There have been many previous studies about the spatial distribution of extreme rainfall events in China. They investigated rainstorm events with different temporal resolutions, and estimated their magnitudes at different return periods using the hydrological frequency analysis approach [21,23,24]. The results found in this study are similar to those in previous studies in terms of the spatial distribution of rainstorms. Comparatively, the advantage of the present study is its simplicity. It just needs to use the index of entropy to analyze daily rainfall data by first removing dry days. The entropy results are determined by statistical characters of rainfall data and closely related to the hydroclimate conditions, and can intuitively reflect the spatial heterogeneity in the occurrence probability of rainstorms over China.

## 4. Conclusions

From the above results, it is concluded that the entropy index can be an effective alternative for indicating the potential occurrence probability of rainstorms. As for China, rainstorm events are more likely to occur in the Pearl River basin, the Southeast River basin, the lower-reaches of the Yangtze River basin, the Huai River basin, and the southwest corner of China. The occurrences of rainstorm events at diverse time scales would have different spatial distributions. Due to the limited observed data, the spatial distribution of the occurrence probability of rainstorms on smaller time scales was not analyzed using the entropy index here. However, considering that the entropy results are solely determined by the statistical characters of rainfall data, and the occurrence of rainstorms on diverse time scales have certain physical cause-relationships, it is thought that the results from this study reflect the spatial heterogeneity of the occurrences of rainstorm events in China. The thresholds of entropy values determined here can also be helpful references for the studies of daily rainstorms in other basins and regions worldwide. More studies with diverse cases can be conducted to further confirm the conclusion, especially for the entropy-AI relationship. The relationship between the entropy values obtained from observed rainfall data and those expected from proper theoretical probabilistic distribution should also be investigated, for predicting the characteristics of the entropy index and analyzing its temporal variability.

## References

[1] Lindell M.K., Prater C.S. (2003). Assessing community impacts of natural disasters. Nat. Hazards Rev..

[2] Hyndman D., Hyndman D. (2016). Natural Hazards and Disasters.

[3] Barros V.R., Field C.B., Dokken D.J., Mastrandrea M.D., Mach K.J., Bilir T.E., Chatterjee M., Ebi K.L., Estrada Y.O., Genova R.C., Intergovernmental Panel on Climate Change (2014). Climate Change 2014—Impacts, Adaptation and Vulnerability: Regional Aspects.

[4] Zhang Z.T. (2007). Characteristics and prevention of mountain torrent disasters in China. China Water Conserv..

[5] Zhang Q., Gu X., Singh V.P., Kong D., Chen X. (2015). Spatiotemporal behavior of floods and droughts and their impacts on agriculture in China. Glob. Planet. Chang..

[6] Sang Y.F., Wang Z., Li Z., Liu C., Liu X. (2013). Investigation into the daily precipitation variability in the Yangtze River Delta, China. Hydrol. Process..

[7] Giupponi C., Mojtahed V., Gain A.K., Biscaro C., Balbi S., Paron P., di Baldassarre G. (2014). Integrated risk assessment of water-related disasters. Hydro-Meteorological Hazards, Risks, and Disasters.

[8] Molle F., Mollinga P. (2003). Water poverty indicators: Conceptual problems and policy issues. Water Policy.

[9] Peterson T.C., Manton M.J. (2008). Monitoring changes in climate extremes: A tale of international collaboration. Bull. Am. Meteorol. Soc..

[10] Zhang X.B., Alexander L., Hegerl G.C., Jones P., Tank A.K., Peterson T.C., Trewin B., Zwiers F.W. (2011). Indices for monitoring changes in extremes based on daily temperature and precipitation data. WIREs Clim. Chang..

[11] Amorocho J., Espildora B. (1973). Entropy in the assessment of uncertainty in hydrologic systems and models. Water Resour. Res..

[12] Sang Y.F. (2013). Wavelet entropy-based investigation into the daily precipitation variability in the Yangtze River Delta, China, with rapid urbanizations. Theor. Appl. Climatol..

[13] Koutsoyiannis D., Montanari A. (2007). Statistical analysis of hydroclimatic time series: Uncertainty and insights. Water Resour. Res..

[14] Chapman T.G. (1986). Entropy as a measure of hydrologic data uncertainty and model performance. J. Hydrol..

[15] Singh V.P. (2015). Entropy Theory in Hydrologic Science and Engineering.

[16] Yalcin C., Rabassa P., Beck C. (2016). Extreme event statistics of daily rainfall: Dynamical systems approach. J. Phys. A Math. Theor..

[17] Sang Y.F., Singh V.P., Wen J., Liu C.M. (2015). Gradation of complexity and predictability of hydrological processes. J. Geophys. Res. Atmos..

[18] Nandargi S., Mulye S.S. (2012). Relationships between rainy days, mean daily intensity, and seasonal rainfall over the Konya catchment during 1961–2005. Sci. World J..

[19] Shannon C.E. (1948). A mathematical theory of communication. Bell Syst. Tech. J..

[20] Arora V.K. (2002). The use of the aridity index to assess climate change effect on annual runoff. J. Hydrol..

[21] Zhai P., Zhang X., Wan H., Pan X. (2005). Trends in total precipitation and frequency of daily precipitation extremes over China. J. Clim..

[22] Yang P., Ren G., Hou W., Liu W. (2013). Spatial and diurnal characteristics of summer rainfall over Beijing Municipality based on a high-density AWS dataset. Int. J. Climatol..

[23] Zhang Y., Xue M., Li B., Chen J., Tao Z. (2016). Spatial characteristics of extreme rainfall over China with hourly through 24-hour accumulation periods based on national-level hourly rain gauge data. Adv. Atmos. Sci..

[24] Zhang Y., Xu Y., Dong W., Cao L., Sparrow M. (2006). A future climate scenario of regional changes in extreme climate events over China using the PRECIS climate model. Geophys. Res. Lett..

